# Therapeutic efficacy of apelin on transplanted mesenchymal stem cells in hindlimb ischemic mice *via* regulation of autophagy

**DOI:** 10.1038/srep21914

**Published:** 2016-02-23

**Authors:** Dong Liang, Dong Han, Weiwei Fan, Ran Zhang, Hongyu Qiao, Miaomiao Fan, Tao Su, Sai Ma, Xiujuan Li, Jiangwei Chen, Yabin Wang, Jun Ren, Feng Cao

**Affiliations:** 1 Department of Cardiology, Chinese PLA General Hospital, Beijing, 100853, China; 2 Department of Cardiology, Xijing Hospital, Fourth Military Medical University, Xi’an, Shaanxi 710032, China; 3 Department of Cardiology, Armed Police Corps Hospital of Shaanxi, Xi’an, Shaanxi 710032, China; 4 Department of Cardiology, the 175^th^ Hospital of Chinese PLA, the Affiliated Southeast Hospital of Xiamen University, Zhangzhou, Fujian 363000, China; 5 Center for Cardiovascular Research and Alternative Medicine, University of Wyoming, Laramie, WY 82071, USA

## Abstract

Mesenchymal stem cells (MSCs)-based therapy provides a promising avenue for the management of peripheral arterial disease (PAD). However, engrafted MSCs are subjected to acute cell death in the ischemic microenvironment. Apelin has been shown to protect bone marrow MSCs against apoptosis although the mechanism of action remains elusive. Here we demonstrated that apelin promoted functional survival of AD-MSCs in ischemic hindlimbs and provoked a synergetic effect with AD-MSCs to restore hindlimb blood perfusion and limb functions. Further *in vitro* studies revealed that a biphasic response in autophagy was induced by apelin in AD-MSCs during hypoxia and hypoxia/reoxygenation (H/R) stages to exert cytoprotective effects against H/R injury. Mechanistically, apelin increased the viability of AD-MSCs via promoting protective autophagy during hypoxia, which was accompanied with activation of AMPK and inhibition of mammalian target of rapamycin (mTOR). To the contrary, apelin suppressed autophagic cell death during reoxygenation, which was accompanied with activation of Akt and inhibition of Beclin1. Our findings indicated that apelin facilitated AD-MSCs-based therapy in PAD, possibly through promoting survival of AD-MSCs by way of autophagy regulation. Our data support the promises of apelin as a novel strategy to improve MSC-based therapy for PAD, possibly through autophagy modulation in MSCs.

Peripheral arterial disease (PAD) remains one of the leading causes of deformity worldwide^1^. Among various therapeutic options for PAD, stem cell-based therapies hold some great promises in the management of PAD^2^. Nonetheless, the therapeutic efficacy has been held back by the poor survival of donor cells^3^. Our previous findings also demonstrated the feasibility of utilizing adipose derived mesenchymal stem cells (AD-MSCs) in the management of PAD courtesy of its multipotency, abundance for harvest and low immunogenicity^4^. However, low survival rate has been identified for the transplanted AD-MSCs within ischemic tissues, hampering the advancement of AD-MSCs in the therapeutics of PAD^4^. To this end, it is pertinent to search for new and novel approaches to promote donor cell survival in order to foster the success of stem cell-based therapy for PAD.

Stem cells drastically differ from somatic cells in their ability of self-renewal and multi-directional differentiation. Due to their relatively long life in the organisms, it is conceived that the cell sweeper autophagy should be indispensable for quality control and maintenance of cellular homeostasis for stem cells^5^,^6^. In spite of the rich knowledge available for somatic cells, the precise role for autophagy in the maintenance and function of stem cells is only beginning to be understood as a result of some recent seminal studies^7^,^8^. Moreover, previous work has been mainly focused on modulation of inflammation and oxidative stress in ischemic microenvironment to improve the survival of engrafted MSCs^9^,^10^. Autophagy, an important regulator of cellular function and survival, was rarely examined and its role was often omitted for the survival of engrafted MSCs in ischemic condition. Accumulating evidence has consolidated for a major role of autophagy, a cellular process involved in protein and organelle degradation, in a variety of physiological processes, including inflammation, oxidative stress, autophagic cell death and immune responses^11^,^12^. Recent evidence also suggested that autophagy may serve as a therapeutic target in the management of ischemia/reperfusion injury^13^. While low levels of autophagy exhibit a cyto-protective role, high levels or sustained autophagy may promote cell injury and irreversible cell death (type 2 programmed cell death)^14^. Not surprisingly, targeting the pro-death and pro-survival routes in the regulation of autophagy has drawn much attention for the management against ischemic diseases.

Apelin (also known as APLN) is a peptide encoded by the *apelin* gene. The *apelin* gene encodes a 77 amino acid preproprotein which can be further cleaved to shorter biologically active fragments, including apelin-12, apelin-13, apelin-16, apelin-17, and apelin-19. Ample of evidence has indicated that the pyroglutamated form of apelin-13 (Pyr-apelin-13) may be the most potent isoform of apelin to serve as the principal biologically active ligand^15^,^16^. The apelin receptor (also known as the APJ receptor) is a G protein-coupled receptor which binds apelin molecule. Apelin is ubiquitously expressed in various organs such as the heart, lung, kidney, liver, adipose tissue, gastrointestinal tract, brain, adrenal glands, endothelium and plasma^17^,^18^. Apelin has been demonstrated to exhibit cyto-protective effects against both cardiac and cerebral ischemic injuries^19^,^20^,^21^. In particular, apelin was found to protect against serum deprivation-induced apoptosis in cultured rat bone marrow mesenchymal stem cells^22^. Given that apelin is known to participate in the regulation of autophagy^23^,^24^, the present study was designed to examine the impact of apelin on the survival of transplanted AD-MSCs in a murine hindlimb ischemia model and the underlying mechanisms involved with a focus on autophagy.

## Results

### Morphology and bioluminescence imaging (BLI) of AD-MSCs^Fluc+GFP+^

**Figure 1:**
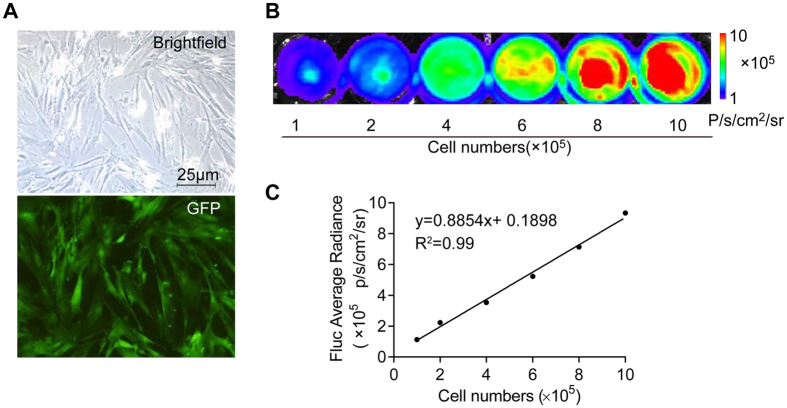
Morphology and BLI images of AD-MSCs *in vitro*. (**A**) Morphology of the third-passage AD-MSCs cultured *in vitro* (up); AD-MSCs is GFP positive (lower panel). (**B**) BLI with different numbers of AD-MSCs. (**C**) A linear relationship is depicted between AD-MSCs numbers and BLI signal intensity; Scale bar: 25 μm.

AD-MSCs^Fluc+GFP+^ cultured in medium displayed a fibroblast-like morphology (Fig. 1A-a). AD-MSCs^Fluc+GFP+^ were positive for eGFP (enhanced green fluorescent protein) under fluorescent microscope (Fig. 1A-b). The stable expression of firefly luciferase (Fluc) was confirmed by bioluminescence imaging (BLI) in AD-MSCs (Fig. 1B). Moreover, cells expressed Fluc reporter gene in a number-dependent fashion as confirmed by BLI. The BLI signal intensity of 1.0 × 10^5^ to 1.0 × 10^6^ AD-MSCs rose gradually from 1.12 × 10^5^ to 9.33 × 10^5^ p/s/cm^2^/Sr. A linear correlation was identified between cell quantities and Fluc signal (correlation coefficient: 0.99; linear regression equation: y = 0.8854x + 0.1898) (Fig. 1C). These data indicated that BLI of Fluc may serve as a reliable tool to monitor viable transplanted AD-MSCs quantitatively *in vivo.*

### Restoration of hindlimb blood perfusion in PAD model following AD-MSCs transplantation and apelin administration

**Figure 2:**
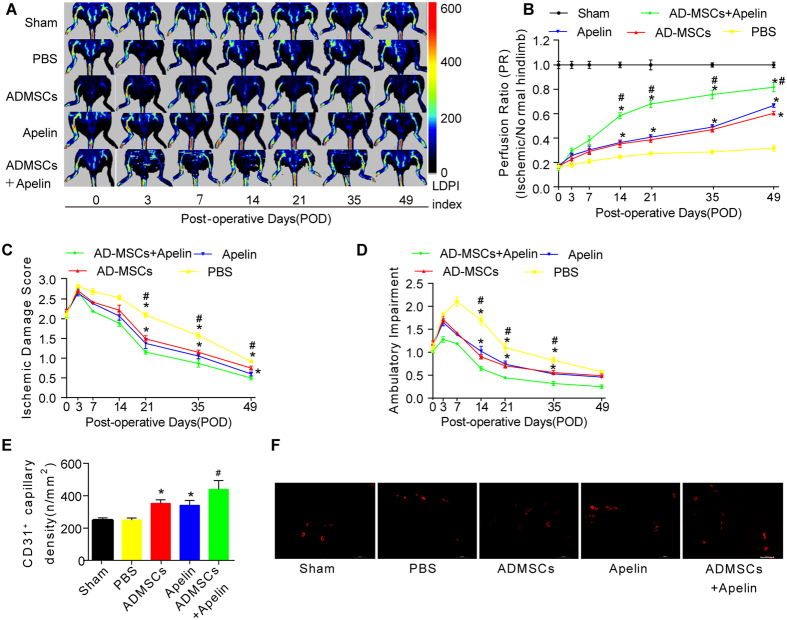
AD-MSCs transplantation and apelin administration promoted hindlimb functional recovery and angiogenesis in the PAD model. (**A,B**) *In vivo* laser Doppler perfusion imaging (LDPI) visualized dynamic changes in hindlimb blood perfusion, which was (**B**) quantified using perfusion ratio (PR), i.e., the ratio of average LDPI index of ischemic (left leg, red arrows, the same to fig. 4A) to nonischemic hindlimbs. Colored scale bar represents blood flow velocity in LDPI index. n = 10. (**C,D**) Cumulative results for functional assessment of ischemic muscle over follow-up are shown graphically as ischemic damage score (**C**) and ambulatory impairment score (**D**), n = 15 for each group. (**E,F**) Representative image and quantitative analysis of the CD31-positive blood vessels within the same-sized regions of adductor muscle section among groups, as assessed by immunofluorescence staining with endothelial marker CD31 (PECAM-1) on POD49. n = 20 random fields. Error bars represent mean ± SD. *P < 0.05 vs. PBS, ^#^P < 0.05 vs. both AD-MSCs and apelin. Scale bar: 100 μm.

*In vivo* laser Doppler perfusion imaging (LDPI) visualized the dynamic changes in hindlimb blood perfusion (Fig. 2A). Perfusion ratio (PR), i.e., the ratio of average LDPI, an index of ischemic to nonischemic hindlimbs, was quantified to evaluate the hindlimb blood perfusion status. As shown in Fig. 2A,B, there was little difference in PR between groups on postoperative day (POD) 0 (*p* > 0.05). PR rose gradually in both groups over the next few days. Mice exhibited a higher perfusion ratio in ischemic hindlimbs in both AD-MSCs and apelin groups compared with that of PBS group (Fig. 2B), with a much more pronounced improvement from combined therapy of AD-MSCs and apelin. PR was significantly higher in AD-MSCs + apelin group than AD-MSCs or apelin group (Fig. 2B).

### Hindlimb functional recovery in PAD model following AD-MSCs transplantation and apelin administration

To reveal hindlimb functional recovery, blind scoring of semi-quantitative assessment of impaired use of murine ischemic limb was performed as described in the method section. As shown in Fig. 2C,D, there was no significant difference in ischemic damage and ambulatory impairment scores between experimental groups on POD 0. Ischemic damage and ambulatory impairment scores gradually dropped over the next few days beyond POD7. Blind scoring depicted that both AD-MSCs and apelin ameliorated ischemic damage and ambulatory impairment compared with that of PBS group (Fig. 2C,D), while combined therapy of AD-MSCs and apelin further improved functional recovery of ischemic hindlimbs (Fig. 2C,D).

### Hindlimb angiogenesis in PAD model following AD-MSCs transplantation and apelin administration

On POD 49, 5 mice from each group were sacrificed for histological staining for CD31. Histological staining analysis indicated that both AD-MSCs and apelin improved the density of CD31-positive vessels compared with that of PBS group (Fig. 2E), while combined therapy of AD-MSCs and apelin further improved the density of the CD31-positive vessels in ischemic hindlimbs (Fig. 2E).

### Survival of engrafted AD-MSCs^Fluc+GFP+^ in murine model of PAD

**Figure 3:**
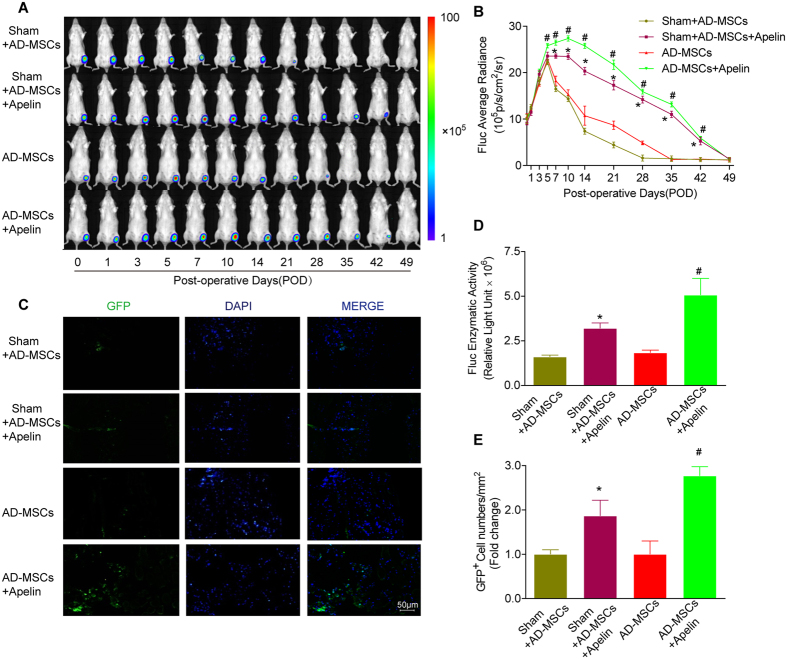
Survival of the engrafted AD-MSCs^Fluc+GFP+^ in murine ischemic hindlimbs following apelin treatment. (**A,B**) Longitudinal BLI spatiotemporally tracked AD-MSCs^Fluc+GFP+^ survival *in vivo* (n = 10 for each group). Quantitative analysis of (**B**) *in vivo* Fluc optical signals demonstrated the progressive death of AD-MSCs^Fluc+GFP+^ following transplantation. Colored scale bar represents BLI radiance intensity in P/s/cm^2^/sr. (**C**) Confocal microscopy of adductor muscle tissue sections with double immunofluorescence staining of GFP (green), and 4′,6-diamidino-2-phenylindole (DAPI, blue) for nuclei as indicated on POD14. (**D**) *Ex vivo* Fluc enzymatic activity on POD14 (n = 5). (**E**) More GFP-positive cells were observed in the apelin treated group than non-apelin treated group (n = 20 random fields).Error bars represent mean ± SD. Scale bars represent 50 μm. *P < 0.05 vs. Sham + AD-MSCs, ^#^P < 0.05 vs. AD-MSCs.

Noninvasive BLI longitudinally revealed the fate of AD-MSCs transplanted into ischemic hindlimbs (Fig. 3A). After initial cell transplantation for 7 days, the BLI signal intensity reached peak. BLI signal exhibited little significant difference among different groups prior to POD3 (*P* > 0.05). However, BLI signal intensity of engrafted AD-MSCs in the Sham + AD-MSCs and AD-MSCS groups experienced a progressive decline in the following 6 weeks, with a decrease in BLI signal from (12.3 ± 0.14) × 10^5^ p/s/cm2/sr and (12.1 ± 0.18) × 10^5^ p/s/cm2/sr respectively on POD0 to (7.4 ± 0.57) × 10^5^ p/s/cm2/sr and (10.72 ± 2.28) × 10^5^ p/s/cm2/sr respectively on POD14, to the background levels on POD49 (Fig. 2B). Apelin treatment improved AD-MSCs survival *in vivo.* In Sham + AD-MSCs + apelin and AD-MSCs + apelin group, donor AD-MSCs exhibited significantly enhanced survival, as evidenced by a remarkably higher BLI signal intensity than their respective non-apelin-treated groups on POD35 (Fig. 3B). This was also confirmed by the significantly higher Fluc enzymatic activity in the apelin-treated groups compared with their respective non-apelin-treated groups on POD14 (Fig. 3D). Moreover, laser confocal microscopy displayed more GFP-positive AD-MSCs within the ischemic tissues in apelin-treated groups on POD14 compared with their respective non-apelin-treated group (Fig. 3C,E).

### The PI3K inhibitor LY294002 abolished the beneficial effects of apelin on ischemic hindlimb recovery and AD-MSCs survival

**Figure 4:**
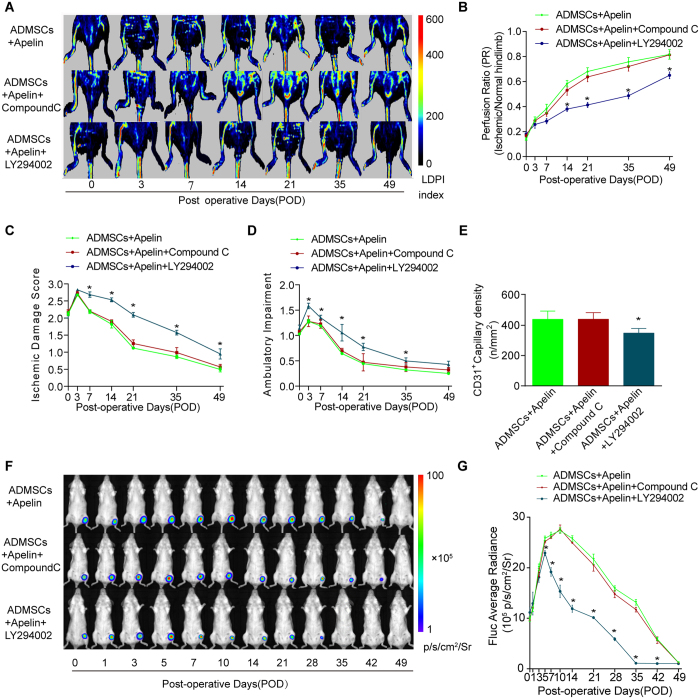
The PI3K inhibitor LY294002 abolished the beneficial effects of apelin on ischemic hindlamb recovery and AD-MSCs survival. (**A,B**) *In vivo* laser doppler perfusion imaging (LDPI) visualized the dynamic changes in hindlimbs blood perfusion, which was (**B**) quantified using perfusion ratio (PR). n = 10. (**C,D**) Cumulative results for functional assessment of ischemic muscle over follow-up are shown graphically as ischemic damage score (**C**) and ambulatory impairment score (**D**), n = 15 for each group. (**E**) Quantitative analysis of the CD31-positive blood vessels within the same-sized regions of adductor muscle section among groups, as assessed by immunofluorescence staining with endothelial marker CD31 (PECAM-1) on POD49. n = 20 random fields. (**F,G**) Longitudinal BLI spatiotemporally tracked AD-MSCs^Fluc+GFP+^ survival *in vivo* (n = 9 for each group). Quantitative analysis of (**G**) *in vivo* Fluc optical signals demonstrated that LY294002 but not compound C abolished the benefical effects of apelin on AD-MSCs survival. *P < 0.05 vs. AD-MSCs + apelin.

We further examined the possible involvement of AMPK and Akt signaling pathways in the pro-survival effect of apelin on AD-MSCs in ischemic hindlimbs. LDPI revealed that the PI3K inhibitor LY294002 but not the AMPK inhibitor compound C abolished the beneficial effects of apelin and AD-MSCs on restoration of hindlimb blood perfusion (Fig. 4A,B). Likewise, LY294002 also abolished the beneficial effects of apelin and AD-MSCs on hindlimb functional recovery as evidenced by the semi-quantitative assessment for ischemic damage and ambulatory impairment scores (Fig. 4C,D). Hindlimb angiogenesis property of AD-MSCs and apelin was reversed by LY294002 as evidenced by histological staining analysis of the CD31-positive vessel density (Fig. 4E). In contrast, these responses were unaffected by the AMPK inhibitor compound C.

Our data further revealed that survival of engrafted AD-MSCs was inhibited by LY294002. BLI assay demonstrated that the PI3K inhibitor LY294002 but not the AMPK inhibitor compound C reversed the pro-survival effect of apelin on AD-MSCs *in vivo*. In AD-MSCs + apelin + LY294002 group, donor AD-MSCs exhibited an early trend of cell death, manifested by remarkably lower BLI signal intensity compared with that from the AD-MSCs + apelin group (Fig. 4F,G).

### Apelin regulated autophagy in AD-MSCs under hypoxia and hypoxia/reoxygenation injury *in vitro*

**Figure 5:**
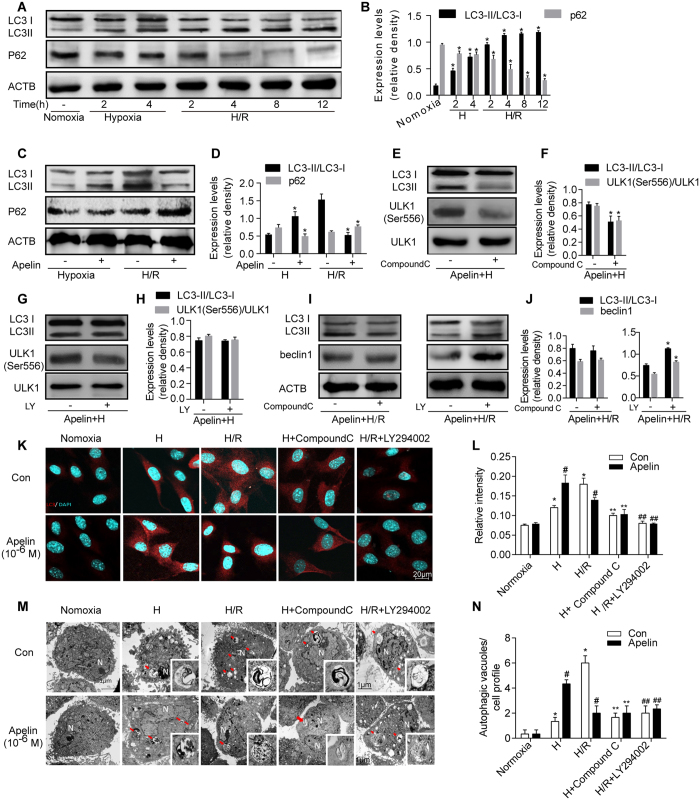
Apelin regulated autophagy in AD-MSCs under hypoxia and hypoxia/reoxygenation injury *in vitro.* (**A**) Representative gel bolts depicting LC3 and p62 protein expression under hypoxia and hypoxia/reoxygenation injury *in vitro* at different time points. (**B**) Quantitative analysis of A. *p < 0.05 compared with normoxia group. (**C**) Representative gel bolts depicting LC3 and p62 protein expression regulated by apelin under hypoxia and hypoxia/reoxygenation injury. (**D**) Quantitative analysis of C. *p < 0.05 compared with respective non-apelin treated group. (**E–J**) Representative gel bolts and respective quantitative analysis of LC3, p-ULK1, and Beclin1 expression. Cells were pretreated with apelin (10^−6^ M) in the presence or absence of AMPK inhibitor compound C(10 μM) or PI3K inhibitor LY294002(20 μM) for 4 h, followed by hypoxia (4 h) or hypoxia (4 h) /reoxygenation (12 h). *p < 0.05 compared with respective non-compound C or non-LY294002 treated group. (**K,L**) Representative immunofluorescence staining of LC3 under confocal microscopy. (**L**) Quantitative analysis of K (n = 10 random fields). *p < 0.05 compared with normoxia group, ^#^p < 0.05 compared with respective non-apelin treated group, **p < 0.05 compared with respective hypoxia group, ^##^p < 0.05 compared with respective H/R group. (**M, N**) Representative Transmission electron microscopy(TEM) images of AD-MSCs after treatment as described in (E). (**N**) Quantitative analysis of M (n = 10 random fields), ^#^p < 0.05 compared with respective non-apelin treated group, **p < 0.05 compared with respective hypoxia group, ^##^p < 0.05 compared with respective H/R group. LY represents LY294002.

To further discern the mechanisms of autophagy behind the pro-survival effects of apelin on AD-MSCs, an *in vitro* hypoxia/reoxygenation (H/R) model was employed to simulate the ischemic hindlimb model *in vivo*. Autophagy level of AD-MSCs was assessed using protein expression of microtubule-associated protein 1 light chain3 (LC3) and SQSTM1 (p62) under hypoxia and H/R at different time points. Both hypoxia and hypoxia/reoxygenation treatments increased the ratio of LC3II/I compared with the normoxia group in all experimental conditions, along with a gradual decrease in p62 protein levels (Fig. 5A,B). Furthermore, apelin (10^−6^ M) treatment promoted autophagy in AD-MSCs under hypoxia (increased ratio of LC3II/I and decreased p62 protein levels compared with the non-apelin treated group (Fig. 5C,D), while the elevated autophagy in AD-MSCs under H/R was abrogated by apelin (10^−6^ M) treatment (manifested as the decreased ratio of LC3II/I and increased p62 protein level compared with non-apelin-treated group) (Fig. 5C,D).

Next, to further explore the regulatory mechanisms of autophagy behind apelin, the AMPK inhibitor compound C (10 μM) or the PI3K inhibitor LY294002(20 μM) was administered to AD-MSCs. Result showed that under hypoxia, the apelin-enhanced autophagy and phosphorylation of ULK1 were reversed by compound C but not LY294002 (Fig. 5E–H). To the contrary, the apelin-suppressed autophagy and Beclin1 expression under H/R were reversed by LY294002 but not compound C (Fig. 5I,J).

To further consolidate apelin-induced autophagy in AD-MSCs, immunofluorescent staining and transmission electron microscope were performed for LC3 and phagophore-autophagosome visualization, respectively. Immunofluorescence of LC3 depicted that LC3 expression was elevated by both hypoxia and H/R compared with normoxia group (Fig. 5K,L). Interestingly, apelin promoted and suppressed autophagy in hypoxia and H/R, respectively (Fig. 5K,L). While compound C abrogated apelin-induced increase in LC3 expression under hypoxia, LY294002 abrogated apelin-suppressed LC3 expression under H/R (Fig. 5K,L). Moreover, transmission electron microscopy (TEM), the most reliable technique for qualitative assessment of autophagy, exhibited similar pattern of autophagy regulation under hypoxia and H/R conditions. Hypoxia and H/R increased number of vacuoles compared with normoxia group (Fig. 5M,N). Consistent with the immunofluorescent staining result, apelin promoted and suppressed autophagy under hypoxia and H/R, respectively (Fig. 5M,N). Likewise, compound C abrogated apelin-induced increase in the number of vacuoles under hypoxia while LY294002 nullified apelin-elicited loss in the number of vacuoles under H/R (Fig. 5M,N).

### Differential autophagy effect in AD-MSCs survival under hypoxia or hypoxia/reoxygenation

**Figure 6:**
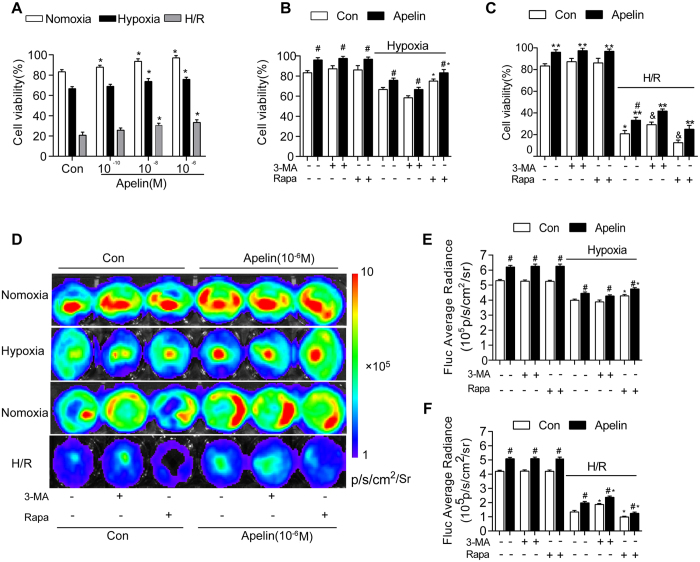
Differential autophagy effect in AD-MSCs survival under hypoxia or hypoxia/reoxygenation. (**A**) Cell viability of AD-MSCs following hypoxia–reoxygenation and different dose of Apelin pretreatment was examined using MTT assay. *p < 0.05 compared with respective control group. (**B,C**) AD-MSCs were pretreated with or without apelin (10^−6^ M) for 24 h. AD-MSCs were then subjected to a 4 hours hypoxia followed with or without a 12 hours reoxygenation (H/R) in the presence or absence of the autophagy inhibitor 3-MA (10 mM) or the autophagy inducer rapamycin (Rapa, 5 μM). Cell viability of AD-MSCs following treatment was examined using MTT assay. ^#^p < 0.05 compared with respective control group without apelin treatment. ^*^p < 0.05 compared with respective control group without inhibitor treatment. (**D**) Representative BLI images of AD-MSCs after treatment as described in (**B**). (**E-F**) Quantitative analysis of Fluc signal in (**D**) (n = 5). ^#^p < 0.05 compared with respective control group without apelin treatment. ^*^p < 0.05 compared with respective control group without inhibitor treatment.

3-(4,5-dimethylthiazol-2-yl)-2,5-diphenyltetrazolium bromide (MTT) assay and *in vitro* BLI were conducted to evaluate cell viability. Hypoxia and H/R significantly decreased cell viability, the effect of which was attenuated by apelin (10^−8^ and 10^−6^ M). In fact, apelin pretreatment significantly increased cell viability in normoxia, hypoxia and H/R conditions (Fig. 6A).

To discern a role for autophagy in apelin-induced AD-MSCs survival, AD-MSCs cells were pretreated with or without apelin (10^−6^ M) for 24 h. AD-MSCs were then subjected to 4-hour hypoxia followed by 12-hour reoxygenation (H/R) in the presence or absence of the autophagy inhibitor 3-MA (10 mM) or the autophagy inducer rapamycin (Rapa, 5 μM). Our data revealed that hypoxia significantly decreased the cell viability, the effect of which was attenuated by apelin. 3-MA significantly enhanced hypoxia-induced AD-MSCs death while mitigating the protective role of apelin. On the other hand, rapamycin effectively rescued hypoxia-induced cell death in a manner reminiscent of apelin. Furthermore, H/R greatly lessened cell survival although with a much less pronounced effect in the apelin-treated group. Interestingly, 3-MA significantly attenuated H/R-elicited cell death in a manner similar to apelin, whereas rapamycin greatly aggravated H/R-induced cell death in both control and apelin groups. These data suggest a likelihood paradoxical role for autophagy in apelin-induced cytoprotection under hypoxia and H/R (Fig. 6B–F).

### AMPK and Akt signaling pathways involved in autophagy during hypoxia and hypoxia/reoxygenation *in vitro*

**Figure 7:**
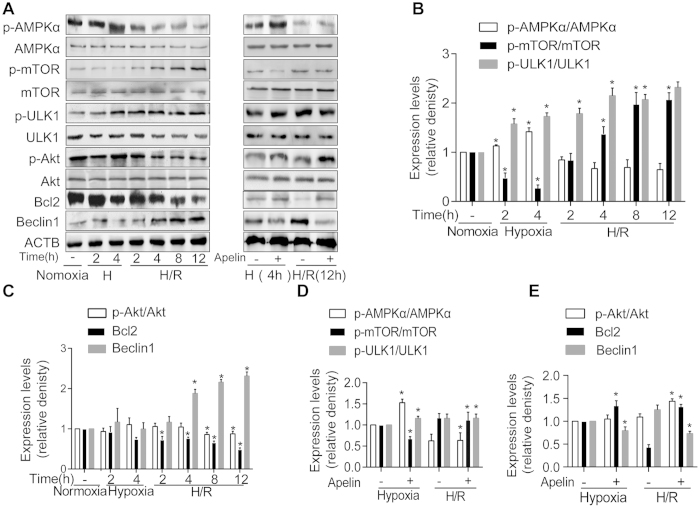
AMPK and Akt signaling pathways in AD-MSCs during hypoxia and hypoxia/reoxygenation *in vitro.* (**A**) Representative gel bolts depicting protein expressions under hypoxia and hypoxia/reoxygenation injury and apelin treatment. (**B,C**) Quantitative analysis of the Western blots. *p < 0.05 compared with normoxia group. (**D,E**) Quantitative analysis of the Western blots. *p < 0.05 compared with respective non-apelin treated group.

**Figure 8:**
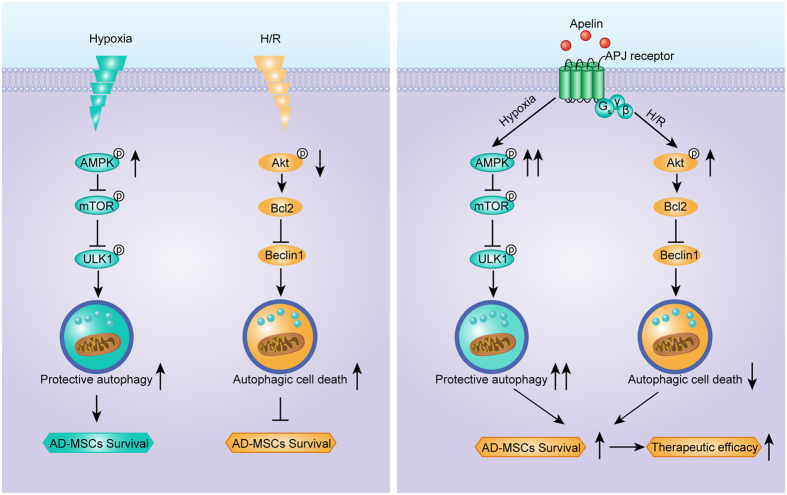
Schematic diagrams depicting apelin regulation of AD-MSCs autophagy under hypoxia and H/R. Hypoxia stimulates AMPK activation and mTOR inhibition in MSCs, leading to a mild elevation of protective autophagy, and promoted MSCs survival in an autophagic aspect (the direct and overall effect of hypoxia is detrimental for MSCs survival), while apelin enhanced AMPK activation, mTOR inhibition, subsequent protective autophagy and AD-MSCs survival in hypoxia phase. On the other hand, H/R leads to a decreased activation of Akt and Bcl2 in MSCs, increasing the level of autophagic cell death, and impairs MSCs survival, while apelin enhanced activation of Akt and Bcl2 in MSCs, leading to suppression of autophagic cell death in H/R. Apelin exhibited the potential to enhance survival of engrafted MSCs via regulation of MSCs autophagy. Thus, apelin may be a potential target for optimizing MSC therapy for PAD.

Mammalian target of rapamycin (mTOR) serves as a critical signaling regulator for autophagy under the positive and negative control of Akt and AMPK, respectively. Protein expression of AMPK, Akt, and mTOR *etc.* was assessed along with autophagy under hypoxia and reoxygenation at different time points. Our data revealed a significant concurrent increase in ULK1 and AMPK phosphorylation under hypoxia in AD-MSCs, which was accompanied by a decline in the phosphorylation of mTOR (Fig. 7A,B). These responses in the phosphorylation of ULK1, AMPK and mTOR were augmented by apelin (10^−6^M) treatment (Fig. 7A,D). Our further findings revealed a significant decrease in Akt phosphorylation and Bcl2 during reoxygenation, which was accompanied by an increase in Beclin1 (Fig. 7A,C). These responses were reversed by apelin (10^−6^M) treatment (Fig. 7A,E). These findings suggested a likely disparate role of autophagy in the apelin-offered cytoprotection against hypoxia and H/R involving AMPK-mTOR-ULK1 signaling in hypoxia phase along with Akt-Bcl2-Beclin1 signaling in reoxygenation phase (Fig. 8).

### Apelin induced eNOS phosphorylation during hypoxia and hypoxia/reoxygenation *in vitro*

To achieve a better understanding of the apelin-elicited cytoprotective effects, we evaluated eNOS phosphorylation in MSCs. Our results shown in supplementary figure revealed that apelin enhanced eNOS phosphorylation in AD-MSCs in hypoxia injury as well as in hypoxia/reoxygenation period (p < 0.05), suggesting a possible role of eNOS in apelin-elicited cytoprotection.

## Discussion

Recently, MSCs have been reported to undergo autophagy which is expected to dictate the therapeutic potential of MSCs in experimental autoimmune encephalomyelitis^25^. Here we demonstrated that apelin-elicited MSCs autophagy contributed to elevated functional survival of AD-MSCs in ischemic hindlimbs and eventually leaded to enhanced AD-MSCs therapeutic potential in experimental PAD. Ischemic context severely impairs the survival and retention of AD-MSCs in targeted sites. Modulation of autophagy by apelin drastically improved the functional survival and therapeutic efficacy of AD-MSCs in experimental PAD. This effect of apelin was associated with enhanced protective autophagy in hypoxia phrase and decreased autophagic cell death in H/R phase. We further revealed that AD-MSCs autophagy during hypoxia phase is mediated through AMPK/mTOR/ULK1 pathway, which was up-regulated by apelin. On the other side of the coin, H/R energized autophagic cell death through an Akt/Bcl2/Beclin1-dependent pathway, which was likely down-regulated by apelin. This biphasic regulation of AD-MSCs autophagy by apelin might account for the enhanced therapeutic potential of apelin in AD-MSCs-based experimental PAD. Our findings suggest that modulation of autophagy in MSCs by apelin may present a novel strategy to improve MSCs therapeutic efficacy in experimental PAD and other ischemic disorders in a much broader context.

Ample preclinical and clinical studies have demonstrated that cell survival and retention is closely related to the outcome of MSC-mediated therapy^25^,^26^. For example, Vrtovec and colleagues revealed that improvement of left ventricular ejection fraction (LVEF) was closely correlated with the cell retention in CD34^+^ peripheral blood mononuclear cells (PBMNCs) mediated therapy for dilated cardiomyopathy (DCM)^25^. Silva and coworkers demonstrated that functional recovery for the heart was partially determined by cell retention in bone marrow mononuclear cells (BMMNCs) mediated therapy for ST elevation myocardial infarction (STEMI)^26^. Moreover, the surviving fraction of donor cells is quite variable in different studies, ranging from 0% to 90%, contributing to the continuing uncertainty for the therapeutic efficacy^27^,^28^. To this end, improving cell survival and retention is pertinent to promote the therapeutic efficacy of MSCs in PAD therapy. Taking advantage of BLI, we longitudinally and spatiotemporally visualized the abbreviated lives of AD-MSCs following their transplantation into murine ischemic hindlimbs *in vivo*, or following H/R insult *in vitro*, which should provide favorable benefits in non-invasive cell tracking of AD-MSCs *in vivo*. Our BLI observation has consolidated that apelin may enhance the functional survival of AD-MSCs in experimental PAD, thus providing a promising measure for future stem cell-based therapy in ischemic diseases.

Previous evidence has indicated the feasibility of apelin in the treatment of PAD. Apelin is reported to be up-regulated following myocardial ischemia to turn on the reperfusion injury salvage kinase pathway, en route to a delay in the mitochondrial permeability transition pore opening and protection against ischemic cardiac injury^29^. On the other hand, apelin was suggested to enhance cardiac neovascularization after myocardial infarction^30^. This seems to fit well with the promising role for therapeutic angiogenesis in the management of PAD. Recent growing evidence has depicted the safety and efficacy of therapeutic angiogenesis using gene and cell therapy^31^. Our previous work demonstrated that AD-MSCs transplantation exhibited great potential in the management of PAD^32^. In our present work, apelin is proved to possess a synergetic effect with AD-MSCs to improve AD-MSCs-mediated limb repair following ischemic hindlimb injury.

A tie has been suggested for apelin and stem cell proliferation. Li and colleagues reported that the apelin/APJ signaling pathway might be involved in hypoxia-induced BMSC (bone marrow stem cells) proliferation^33^. Their findings revealed a possible role for apelin in the processes of BMSC proliferation through the Akt/GSK3β CyclinD1 pathway^34^. Similarly, apelin has been reported to promote hypoxia-induced proliferation of endothelial progenitor cells and to increase myocardial progenitor cells following myocardial infarction^35^,^36^. In fact, apelin possessed mitogenic effects on a wide variety of cell types and was capable of stimulating the growth or proliferation of many different cell types including human umbilical vein endothelial cells^37^, human vascular smooth muscle cells^38^. Consistent with these reports, our current findings revealed a rather crucial role for apelin in the proliferation and survival of AD-MSCs subjected to hypoxia and hypoxia/reoxygenation injury, possibly through autophagy regulation in AD-MSCs.

A large amount of evidence supported that autophagy was closely related to cell survival or cell death^39^. On one hand, agents capable of inducing autophagy have been found to be cytoprotective, such as rapamycin and statins^7^,^40^. On the other hand, autophagy may also promote cell death^41^. It is generally conceived that moderate autophagy may be cytoprotective while excessive autophagy may result in autophagic cell death^14^. Our data showed that apelin treatment facilitates autophagy in hypoxia phase and inhibits autophagy in reoxygenation phase. Likewise, inhibition of autophagy abrogated cytoprotective properties of apelin in AD-MSCs during hypoxia, while inhibition of autophagy promoted the pro-survival capacity of apelin during reoxygenation. Therefore, although hypoxia-induced autophagy in AD-MSCs may be protective in general, it turned out to be rather detrimental during reoxygenation. Coincidentally, apelin is capable of enhancing the protective autophagy in hypoxia, while suppressing the autophagic cell death in H/R. A combination of these properties appeared to be responsible for the ultimate protective effect of apelin against H/R injury.

Our data further depicted a possible role for the AMPK-mTOR-ULK1 signaling cascade in autophagy induction en route to the beneficial effects of apelin during hypoxia. The two protein complexes AMPK and mTORC1 are known to counter- regulate the autophagy inducing complex ULK1/2-Atg13-FIP200^42^,^43^,^44^,^45^. Under the low-energy conditions, AMPK positively regulates autophagy through inhibition of mTORC1. AMPK activation releases the inhibitory regulation of mTORC1 on the ULK1/2-Atg13-FIP200 complex, especially on ULK1/2 kinase activity^45^. The AMPK/mTOR/ULK1 pathway represents an attractive target for therapeutic treatment of autophagy^46^. In our hands, AMPK/mTOR/ULK1 pathway was up-regulated by apelin and may mediate protective autophagy under hypoxia *in vitro*. However, our *in vivo* data suggested that inhibition of AMPK/mTOR/ULK1 pathway using compound C failed to affect AD-MSCs survival as well as the combined therapeutic efficacy of AD-MSCs and apelin in experimental PAD. One plausible explanation may be that our *in vivo* experimental PAD model was more likely to be an ischemia/reperfusion process. After an initial short-period of ischemia, reperfusion process gradually took over (as evidenced by laser Doppler perfusion imaging for blood reperfusion). Thus the reperfusion process is deemed to be more critical than the initial short-period of ischemia in experimental PAD therapy.

Our study also demonstrated the Akt activation, Bcl2 activation to engage Beclin1 inhibition and suppressed autophagy en route to the beneficial action of apelin during reoxygenation, when AMPK is no longer active. Phosphorylation/activation of Akt kinase is known to regulate Bcl2, the Akt-Bcl2 pathway represents an important antiapoptotic signaling^47^. Furthermore, Pattingre and colleagues reported that Bcl-2 antiapoptotic proteins inhibit Beclin 1-dependent autophagy, which is compatible with cell survival^48^. Besides, Matsui and coworkers reported that suppressed autophagy via 3-MA or Beclin 1 knockout during reperfusion was accompanied by pronounced reduction in infarct size and apoptosis following simulated I/R^49^. In our present work, apelin likely suppressed autophagic cell death through the Akt/Bcl2/Beclin1 pathway in H/R, thus promoting AD-MSCs survival under H/R injury. Our *in vivo* data also favored the notion that the Akt pathway seemed to be more important than AMPK pathway in apelin-improved functional survival and therapeutic efficacy of AD-MSCs. This may be attributed to our experimental PAD model as mentioned above. Taken together, the orchestration between AMPK and Akt signaling for autophagy seems to play a pivotal role in survival and function of AD-MSCs. These findings should help to shed some lights towards a better understanding of the protective role for apelin against I/R injury in ischemic hindlimbs.

Despite the clinical relevance of our findings, our study suffers from a number of limitations. For instance, although reporter gene imaging can be applied as a powerful tool for *in vivo* tracking of surviving stem cells, this technique is still limited within the laboratory^50^. However, the translation of reporter gene imaging from bench to beside is of great significance for the progress of stem cell therapy^50^. Besides, the autophagy activity of engrafted AD-MSCs *in vivo* was not longitudinally evaluated for apparent technological reasons. *In vitro* H/R model may not fully mimic the ischemia/reperfusion microenvironment *in vivo*.

In summary, our work demonstrated a beneficial role of apelin in promoting the functional survival and therapeutic efficacy of AD-MSCs in stem cell based therapy for PAD. Apelin was shown to protect AD-MSCs against H/R injury, possibly via an AMPK-dependent induction of autophagy during hypoxia and an Akt-dependent suppression of autophagy during reoxygenation. This finding may warrant the consideration of a prospective clinical trial to evaluate the potential therapeutic impact of combined apelin-ADMSC treatment for patients with PAD to the conventional therapy.

## Methods

### Animals

Fluc^+^-eGFP^+^ double transgenic mice (Tg [Fluc-egfp]) were bred on a FVB/N background, which could constitutively express firefly luciferase (Fluc) and enhanced green fluorescence protein (eGFP) in all tissues and organs, and were used for AD-MSCs isolation^4^,^51^. Syngeneic female FVB mice with the same genetic background as FVB mice (wide type, n = 360, 10-week-old, 20–25 g, SPF) were used for PAD model. This setting of cell recipients and cell donors should greatly minimize the immunogenicity raised by allogeneic MSCs^52^. All procedures were performed in accordance with the National Institutes of Health Guidelines on the Use of Laboratory Animal. Experimental protocols and animal care methods were approved by the Fourth Military Medical University Committee on Animal Care.

### Isolation, Culture, and Identification of AD-MSCs^Fluc+GFP+^

AD-MSCs^Fluc+GFP+^ were isolated from Fluc + -eGFP + double transgenic mice and expanded using our previously described procedure with minor modifications^4^,^53^. Cultured AD-MSCs were identified for immunophenotype and multipotency using flow cytometry and chemical induction as we have previously described^4^.

Reporter gene imaging was performed to determine the stable expression of firefly luciferase (Fluc) in AD-MSCs. AD-MSCs of different quantities ranging from 1 × 10^5^ to 10 × 10^5^ were seeded into 96-well plates, suspended in 500 μl phosphate-buffered saline(PBS), incubated with reporter probe D-luciferin (150 ng/μl, 88293, Invitrogen), and then imaged using a charge-coupled device (CCD) camera within Xenogen Kinetic *In vivo* Imaging System (IVIS, Caliper Life Sciences), with the following parameters: Binning: 4, F/Stop: 1, and Exposure time: 1 minute. Peak signal intensity was expressed in average radiance unit (photons/second/cm^2^/steridian, P •s^−1^cm^−2^sr^−1^) from a fixed-area region of interest (ROI). LivingImage 4.2 software (Caliper, MA, USA) was used for imaging analysis.

### H/R injury *in vitro*

Cultured AD-MSCs of third passage were stimulated with H/R injury as previously described^51^. Briefly, AD-MSCs were plated in 24-well plates (5 × 10^4^ cells per well). Twenty-four hours later, AD-MSCs were administrated with PBS, cultured in Hanks buffer(GIBCO,14025076). Then, different doses of apelin (ab141010, abcam, 10^−10^, 10^−8^, 10^−6^) were added into respective wells for 6 hours. After that, AD-MSCs were incubated in an anoxic chamber (95% N 2 /5% CO_2_) (Thermo) at 37 °C for indicated period of time and subsequently moved or not moved(hypoxia only) into a normoxia incubator (95% air/5% CO_2_) at 37 °C for for indicated period of time, with refreshed cultured media. In the control group, AD-MSCs were maintained at normoxia (95% air, 5% CO_2_) for equivalent periods.

### Assessment of cell viability

Cell viability of cultured AD-MSCs were evaluated by both 3-(4,5-dimethylthiazol-2-yl)-2,5-diphenyltetrazolium bromide (MTT) assay and *in vitro* BLI. Briefly, cells were plated in 96-well plates at 1 × 10^4^/well. After hypoxia, H/R or normoxia treatment, cells from each group were harvested and incubated with 10 μL MTT (KA1606, abnova, 5 g/L) for 4 h. After that, the incubation medium was removed and formazan crystals were dissolved in 150 μL dimethyl sulphoxide (DMSO, BY12065, Sigma). The absorbance was determined at a wavelength of 490 nm.

For *in vitro* BLI, AD-MSCs were plated in 24-well plates (5 × 10^4^ cells per well) for 24h, followed by H/R or normoxia treatment, after that, firefly luciferase (Fluc) in AD-MSCs were detected by a charge-coupled device (CCD) camera within Xenogen Kinetic *In vivo* Imaging System (IVIS, Caliper Life Sciences) as described above.

### PAD Model and Cell Delivery

FVB mice (n = 360) were randomized into nine groups (n = 40 each, matched for weight): (1) Sham group; (2) Sham + AD-MSCs group; (3) Sham + AD-MSCs + apelin group; (4) PAD + PBS group(PBS); (5) PAD + AD-MSCs group(AD-MSCs); (6) PAD + apelin group(apelin); (7)PAD + AD-MSCs + apelin group(AD-MSCs + apelin); (8) PAD + AD-MSCs + apelin + Compound C(AD-MSCs + apelin + Compound C), (9) PAD + AD-MSCs + apelin + LY294002(AD-MSCs + apelin + LY294002). For the PAD model, unilateral hindlimb ischemia was induced by ligating and excising the left femoral artery with all superficial and deep branches for all groups except Sham. Surgical procedure for hindlimb ischemia is as previously described with minor modifications^5^. Sham-operated mice received incision without artery ligation or PBS treatment. Mice in the Sham + AD-MSCs, Sham + AD-MSCs + apelin, AD-MSCs, AD-MSCs + apelin, AD-MSCs + apelin + Compound C and AD-MSCs + apelin + LY294002 groups were subjected to AD-MSCs (1.0 × 10^7^) delivery. Cells were suspended in 30μl PBS and cautiously injected into the left adductor muscle using a 29-gauge insulin syringe (324910,BD Biosciences).Sham + AD-MSCs + apelin, apelin, AD-MSCs + apelin, group animals were administered apelin-13 (1 mg /kg per day for consecutive 14 days from POD0, ab141010, abcam) via adductor injection, AD-MSCs + apelin + Compound C and AD-MSCs + apelin + LY294002 group animals were administered Compound C (50 nM/kg per day for consecutive 14 days from POD0, 171260-10MG, EMD Millipore) or LY294002 (200 nM/kg per day for consecutive 14 days from POD0, 9901S, Cell Signaling Technology), respectively, via adductor injection, while PBS group animals received PBS only, without AD-MSCs.

### *In vivo* BLI for AD-MSCs tracking

*In vivo* BLI was performed to track the survival of engrafted AD-MSCs. Mice were anesthetized and intraperitoneally injected with 150 mg/kg D-luciferin(88293, Invitrogen). Using IVIS, images were acquired at 3-minute intervals until the peak signal was observed. Fixed-area region of interests(ROIs) were created over left hindlimbs, and photons emitted from the ROIs were quantified by P s^−1^cm^−2^sr^−1^ using Living Image software (Caliper, MA, USA). Animals were longitudinally imaged at 0,1,3,5,7,10,14,21,28,35,42,49 days post operation.

### *Ex vivo* Luciferase Assay

Left adductor muscle tissues were removed from sacrificed mice on POD14, homogenized in PBS containing a protease inhibitor cocktail (B14001, Selleck), and lysed with 1 × PLB (passive lysis buffer). After centrifugation at 15,000 rpm for 10 minutes at 4 °C, the supernatant was collected and then measured using the Luciferase Assay System for Luciferase activity.

### Serial Laser Doppler Perfusion Imaging of Hindlimbs

Laser Doppler perfusion imaging (LDPI) was used to serially monitor the blood perfusion recovery of the ischemic hindlimbs. Briefly, mice were placed on a 37.4–38.0 °C heating pad to minimize temperature variation and then imaged using an analyzer (PeriScan-PIM3 Perimed AB, Sweden). The blood flux was quantified using perfusion ratio [PR, ratio of average LDPI index of ischemic to non-ischemic (contralateral, self-control) hindlimb] by LDPI win 3.1.3 (Perimed AB).

### Blind scoring for murine ischemic damage and ambulatory impairment

Semiquantitative assessment of impaired use of murine ischemic limb was performed as previously described at different time points^54^. Briefly, ischemic damage score were set as: 3 = dragging of foot, 2 = no dragging but no plantar flexion, 1 = plantar flexion, and 0 = flexing the toes to resist gentle traction on the tail. Ambulatory impairment score were set as: 0 = no difference from the right hindlimbs, 1 = mild discoloration, 2 = moderate discoloration, 3 = severe discoloration or subcutaneous tissue loss or necrosis, and 4 = any amputation. Amputation was defined as necrosis beyond the level of toes, including loss of ischemic lamb or loss of knees. All assessments were performed and averaged by 3 blinded and independent investigators.

### Transmission electron microscopy

After indicated treatment, cultured AD-MSCs were collected and placed in a tube and centrifuged at 1,500 rpm for 10 min and the supernatant was carefully absorbed. The cells were then fixed with 3% glutaraldehyde(sc-358787, santacruze) and 1% osmium tetroxide (sc-206008, santacruze) for 24 h. Following rinsing with PBS for 30 min, the samples were dehydrated with ethanol and isopropanol, embedded in epoxy resin and prepared under a dissecting microscope. An ultrathin sectioning machine (Leica EM UC6, Leica Microsystems, Manneim, Germany) was used to prepare the 1-μm sections, then the samples were double stained with uranyl acetate and lead citrate. Ultrathin sections were observed using TEM (JEM-1200EX, JEOL Ltd., Tokyo, Japan). Images were captured and 10 randomly selected fields of vision from each group were used to quantify the area of the autophagosomes to the total cytoplasmic area.

### Immunofluorescent staining

Immunofluorescence was performed to detect LC3 expression in AD-MSCs as well as AD-MSCs marker eGFP and angiogenesis marker CD31 in frozen sections of left adductor muscle. Briefly, frozen sections of left adductor muscle or cells were sequentially fixed within cold acetone for 10 min, washed three times with PBS containing 0.3% Triton X-100 and blocked with goat serum for 30 min at room temperature. Antibodies against LC3 (1:100; Cell Signaling Technology, #4108), GFP(1:2000, abcam, ab6556) and CD31(1:50; abcam, ab28364) was incubated overnight at 4 °C, followed by detection with corresponding fluorescent secondary anti-bodies (Santa Cruz Biotechnology, Santa Cruz, CA) for 1 h at 37 °C. Nuclei were counterstained with 49, 6-diamidino-2-phenylindole (DAPI, 4083S, Cell Signaling Technology). Slides were photographed by confocal microscope (FluoView-FV1000, Olympus, Japan). Image-Pro Plus 4.5 software (Media Cybernetics, Silver Spring, USA) were used to analyze fluorescence intensity.

### Western blotting assay

After respective treatment, AD-MSCs were washed and scraped using lysis buffer[25 mM Tris-HCl (pH 7.4), 150 mM NaCl, 2 mM EDTA, 1% Triton-X-100, 1% sodium deoxycholate, 0.1% sodium dodecyl sulfate and protease inhibitor cocktail]. Samples consisting of 50 μg total protein were loaded onto an SDS-PAGE gel(P0012AC, Beyotime) and transferred electrophoretically to nitrocellulose membranes (LC2000, invitrogen). After blocking with 5% bovine serum albumin in PBS, the membranes were incubated with the appropriate primary antibody against LC3 (#4108, Cell Signaling Technology,1:1000), p62 (ab56416, abcam,1:500), p AMPKα(ab13348, Anti-AMPK alpha 1 [phospho T183] + AMPK alpha 2 [phospho T172], abcam,1:1000), AMPKα(ab80089, abcam,1:1000), p-mTOR(#2971, Ser2448, Cell Signaling Technology, 1:2000), mTOR(#2972, Cell Signaling Technology, 1:2000), p-ULK1(#5869,Ser555, Cell Signaling Technology, 1:1000), ULK1(#8054,Cell Signaling Technology, 1:1000), p-Akt1(ab81283,Ser473,abcam,1:5000), Akt(ab32505, abcam,1:5000), Bcl-2(ab692, abcam,1:500), Beclin-1(ab16998, abcam,1:500) or ACTB (TA-09, Zhongshan Jinqiao Biotechnology Co.,1:2000) at 4 °C overnight. The next day, the blots were washed and incubated in the appropriate secondary antibodies(ab6721, ab6789, abcam) at room temperature for 1 h. Subsequent to being washed, the blots were developed and gray scale scanning (iBox Scientia 500/600, UVP, Upland, CA, USA) was performed. The expression levels of LC3, p62, Bcl-2 and Beclin-1 proteins were normalized to ACTB(β-actin). Quantitative analysis was performed using QuantiOne imaging software (Bio-Rad, USA) to assess the integrated optical density (IOD) of each band.

### Statistical analysis

All analyses were performed with SPSS 20.0 software (SPSS Inc., Chicago, IL, USA). The measurement data are presented as mean ± standard deviation (SD) and the multi-group comparisons were made with a one-way factor analysis of variance, followed by Dunnet’s post hoc test. Data expressed as proportions were assessed with a Chi square test. Values of P < 0.05 were considered to indicate a statistically significant difference.

## Additional Information

**How to cite this article**: Liang, D. *et al.* Therapeutic efficacy of apelin on transplanted mesenchymal stem cells in hindlimb ischemic mice *via* regulation of autophagy. *Sci. Rep.* **6**, 21914; doi: 10.1038/srep21914 (2016).

## Supplementary Material

Supplementary Figure
