# Supplementary material for: Therapeutic efficacy of apelin on transplanted mesenchymal stem cells in hindlimb ischemic mice via regulation of autophagy
Source: Sci Rep. 2016 Feb 23;6:21914. doi: 10.1038/srep21914 (PMC4763210; doi:10.1038/srep21914)

Therapeutic efficacy of apelin on transplanted mesenchymal stem cells in hindlimb ischemic mice *via* regulation of autophagy

Dong Liang[1,3#], Dong Han[1,2#], Weiwei Fan[1,4], Ran Zhang[1], Hongyu Qiao[2], Miaomiao Fan[2], Tao Su[2], Sai Ma[2], Xiujuan Li[2], Jiangwei Chen[2], Yabin Wang[1], Jun Ren*[5], Feng Cao*[1,2].

[1] Department of Cardiology, Chinese PLA General Hospital, Beijing, 100853, China

[2] Department of Cardiology, Xijing Hospital, Fourth Military Medical University, Xi'an, Shaanxi 710032, China

[3] Department of Cardiology, Armed Police Corps Hospital of Shaanxi, Xi'an, Shaanxi 710032, China

[4] Department of Cardiology, the 175th Hospital of Chinese PLA, the Affiliated Southeast Hospital of Xiamen University, Zhangzhou, Fujian 363000, China

[5] Center for Cardiovascular Research and Alternative Medicine, University of

Wyoming, Laramie, WY 82071, USA

[#] These authors contributed equally to this work and should be considered as co-first authors.

*Correspondent author:

Jun Ren, MD, PhD

Center for Cardiovascular Research and Alternative Medicine, University of Wyoming, E-mail: jren@uwyo.edu;

Feng Cao, MD, PhD

Department of Cardiology, PLA General Hospital, Beijing, 100853, China, E-mail: wind8828@gmail.com, Tel:+86-10-55499138

Email addresses: DL: liangdongld@gmail.com; DH: handong123566@163.com; WWF:820723047@qq.com;RZ:bjzhangran@126.com;HYQ: qiaohongyu.1990@163.com;MMF:yuehaixiang1986@163.com;TS: utao2001@gmail.com; SM: masaimacey@gmail.com; XJL: lixiujuan0716@126.com; JWC:cjwfmmu@gmail.com;YBW: scottss0415@gmail.com; JR: jren@uwyo.edu；FC： wind8828@gmail.com

Supplementary Figure Legends

Supplementary Figure: Determination of eNOS phosphorylation in MSCs. To achieve a better understanding of apelin-elicited cytoprotective effects, eNOS phosphorylation was assessed in MSCs. AD-MSCs were pretreated with or without apelin (10^{-6} M) for 24 hours. AD-MSCs were then subjected to a 4-hour hypoxia(H) followed with or without a 12-hour reoxygenation (H/R). eNOS phosphorylation was then determined using western blot. Presented values are means ± SD. N=3 /group. Con, Control, *p＜0.05 among groups.

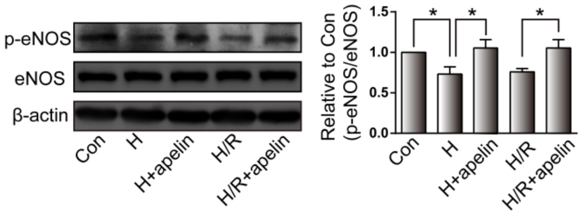

Supplement: Supplementary Figure [file srep21914-s1.pdf]
